# Essential Crimes? Essential Punishments? Rethinking Essentiality in the Midst of the COVID-19 Pandemic

**DOI:** 10.1007/s10612-021-09564-2

**Published:** 2021-04-03

**Authors:** Valeria Vegh Weis, Brittany Magnin

**Affiliations:** 1 Freie Universität Berlin Law School, Berlin, Germany; 2 Buenos Aires University Law School, Buenos Aires, Argentina

## Abstract

The phrases, “essential businesses” and “essential jobs,” emerged at the beginning of the COVID-19 pandemic, raising questions about and reflecting concerns over which goods, services, and workers were necessary to prevent societal collapse. In an attempt to continue to probe “essentiality,” this article coins the term “essential crimes” to refer to those socially injurious acts and omissions that are part and parcel of a global neoliberal capitalist order, and that are, therefore, vital to keep the socioeconomic system running. In other words, if keeping humans alive in the midst of the COVID-19 pandemic required supermarkets and hospitals to remain open (“essential business and jobs”), maintaining the existing socioeconomic system and ensuring that the powerful remained powerful required harmful acts and omissions by states and corporations—what we refer to as “essential crimes.” This article sheds light on how the COVID-19 pandemic has helped illuminate just how *essential* these crimes and harms are to the perpetuation of the status quo by the powerful. In addition, this article encourages us to consider which punishments, if any, are vital to a well-ordered society, and it demands that we rethink whether prison is an “essential punishment” for ensuring public safety.

## Introduction

The COVID-19 pandemic invites us to think broadly about what is *essential* in our individual lives. Governments around the world have had to face the challenging decision of identifying what and who are really essential for our societies in terms of personnel. For example, without doctors, nurses and other healthcare professionals, public health would suffer and morbidity and mortality would increase. In the same vein, only specific companies and stores have been deemed essential businesses and have been allowed to remain open—and, in some instances (such as supermarkets), for longer hours. Indeed, for much of the world, supermarkets are necessary for people to obtain food. Meanwhile, guidelines and orders regarding social distancing and sheltering-in-place have forced us all to think about our essential relationships.

This article explores the concept of *essentiality* in the context of our criminal justice system by putting forth two ideas: “essential crimes” and “essential punishments.” At first blush, the notion of an “essential crime” might seem odd or even oxymoronic. Usually, crime is something that societies want to *prevent* or *reduce*. How can an act or omission that causes harm be important or necessary for a society? But as we intend to argue, certain acts and omissions, while injurious to many, are integral to the success of powerful sociopoliticoeconomic entities. Thus, they are *essential* to the maintenance of an order that benefits these elites: they are “essential crimes” for the powerful to (commit to) remain powerful. And during the COVID-19 pandemic, as we will explain, many of these acts and omissions—many of these “essential crimes” by states and corporations—were laid bare.

The notion of “essential punishments” is a bit more straightforward in that it serves as a heuristic device for inquiring which penalties and sanctions are really necessary for public safety. The pandemic allowed for the early release of many individuals serving time in jails and prisons, and as we argue, provided us with an opportunity to consider not only the nature, type, and extent of certain punishments, but *who* should be punished and *for what*.

Part I of this article develops the concept of “essential crimes” by situating it in the criminological literature on social harms and crimes of the powerful. Part II explores different state crimes, corporate crimes, and state-corporate crimes committed during the COVID-19 that were or have been particularly *essential* to powerful entities. Part III analyzes the notion of “essential punishment” and Part IV concludes the article with some final reflections. Overall, the argument of the article is that it is *essential* (absolutely necessary, extremely important) to identify and reflect upon the extensive social harms committed during the COVID-19 pandemic, to understand how those harms are integral to the existence and success of powerful entities, and to rethink the priorities of our criminal justice systems, which keep downplaying the crimes of the powerful and perpetuating certain antiquated forms of punishment.

## “Essential Crimes”?: Crimes Essential to the Powerful; Crimes Essential for Us to Study

Many of the workers that we consider “essential” (e.g., delivery employees, supermarket cashiers, farmworkers, medical care staff) are not employed directly by the companies for whom they work but by recruiting services; they often do not have a contract and may be paid under the table. The current economic system has contributed to the emergence of these precarious, underpaid, and temporary jobs, particularly in the service sector (Wacquant and Standing 2011; Young 2007). This is a global trend but the situation in the United States (US) is particularly critical. For example, the largest employer in the US, Walmart, pays its employees a low hourly wage, often leaving its workers with no option but to rely on food stamps and Medicare (USA Today 2019).

Even though these “essential workers” produce the food we need, deliver the goods we order, and care for us when ill, all too often, they are treated by corporate entities in ways that do not allow them to meet their own basic needs. As consumers, we may be complicit in that we make purchases and accept deliveries from workers without inquiring whether the individuals providing us with such “essential” goods are being paid a living wage—or even a subsistence wage. In short, we measure “essentiality” according to an ontological reality: if a person provides a service or product that we deem “essential,” then that person is an “essential worker.”

Criminologists often follow a similar logic. They study and focus on acts and omissions that are labeled “crimes” because they are categorized as “crimes.” But as Schwendinger and Schwendinger (1970) and others (e.g., Hillyard et al. 2004; Presser 2013) have argued, we should focus on what behaviors are *harming* society, regardless of whether or not they appear in criminal statutes. Many harmful behaviors are, indeed, not criminalized—particularly those perpetrated by the state or powerful actors—because such powerful entities are the ones making the laws and do not tend to criminalize their deviant behavior (or enforce violations thereof).

All of us would probably agree that the doctor is more “essential” than the football player, at least in times of pandemic. Following the same logic, we might also agree that middlemen mask brokers making massive profits by depriving the population of access to facial protection and putting people at risk of contracting a virus (in this case, COVID-19) should be regarded as committing a greater wrong, at least in times of pandemic, than the person stealing our cell phone (see McSwane 2020; Willber et al. 2020; Salman et al. 2020).

In the next part, we offer various examples of behaviors that have endangered the lives and physical integrity of people around the world, but which have not been appreciated, much less prioritized, by the criminal justice system. These are not actions committed by isolated individuals in the streets, but reflect the so-called “crimes of the powerful.”

What are “crimes of the powerful”? Barak (2015: 104) offers the following definition:

[Crimes of the powerful are] the crimes of the economy or the crimes of capital accumulation and reproduction. Within the prevailing interests and relations of global capital, these crimes include those institutionalized political and economic arrangements that structurally routinize harm, injury, and victimization … refer to those transgressions that simply normalize victimization as “the costs of doing business” and as “collateral damages.”

Barak (2015:105) adds that the lack of criminalization of these behaviors (or the lack of prosecution of individuals who commit such harms when they are proscribed by law) is dismissed because of the alleged necessity of capital accumulation and enhancing the interests of the capitalist state.

This phenomenon can be understood by considering the notion of “criminal selectivity,” which refers to the ways in which the criminal justice system prioritizes the enforcement of violations of certain laws while ignoring others. “Criminal selectivity” also describes the overly punitive treatment of those behaviors committed by individuals that are in vulnerable positions because of their class, gender, race, sex, and sexual orientation (“over-criminalization”) and the absence or minimization of the punitive treatment of behaviors committed by those individuals in positions of power (“under-criminalization”) (Vegh Weis 2017). “Criminal selectivity” also involves a second dimension: “under-criminalization” of acts and omissions by the powerful often harm those who are the most vulnerable members of society, resulting in what can be considered “selective victimization” (Barak 2015).

The next part explores the current situation in the US during the COVID-19 pandemic and utilizes the concepts of “crimes of the powerful,” “criminal selectivity,” “over-criminalization,” “under-criminalization,” and “selective victimization” to analyze what should be deemed the essential focus of the criminal justice system during the pandemic. In particular, we make the argument that state crimes, crimes of globalization, state-corporate crimes, and corporate crimes have been “essential crimes” for those who have committed them and that it is *essential* that criminologists and the criminal justice system devote more attention to them.

## “Essential Crimes” and COVID-19 in the US

### State Crimes of Omission

When dealing with egregious crimes and harms, some key examples are those in which a state, charged with the well-being of its citizens, is, in turn, the one responsible for causing the greatest harms. Barak (1991) defines “state crimes” as those acts that bring material, physical, or social harm to its people resulting from the actions or consequences of government policy mediated through the practice of state agencies, whether these harms are intentional or unintentional. Rothe (2013: 27), in turn, describes “state crime” as “actions that violate domestic or international public law or cause serious social and personal harm through acts of omission or commission, varying from genocide, war crimes, crimes of aggression, illegal use of unmanned aerial vehicles, to racial segregation, immigration policies, and lack of social support systems for the most vulnerable populations.” Notably, the concept of “state crime” has been enriched by Ward’s (2004: 86) notion of “state harm,” defined as “an invasion by a state agency of any person’s basic welfare interests, whether such invasion is justified or not,” including lawful imprisonment.

With these definitions in mind, we can see that state agencies might perpetrate state crimes through various acts or omissions. Kauzlarich and Mullins (2003: 245) assert: “[d]ue to the nature of trust involved in a state as a social institution, when a state or state agency refuses or fails to act responsibly and such action or inaction generates harm, we can call this a crime.” These are known as “crimes of omission” and, once again, are not necessarily proscribed by law but are part of the general expectations of the social contract.

Interestingly, state crimes of omission might be characterized as either “explicit acts of omission,” which occur “when the state disregards unsafe and dangerous conditions, when it has a clear mandate and responsibility to make a situation or context safe” (Kauzlarich and Mullins 2003: 249), or “implicit acts of omission,” which refer to “state complicity in these wider, but nonetheless injurious structural relationships and practices” (Kauzlarich and Mullins 2003: 250), including structural sex, racial, gender and class biases. In the context of the COVID-19 pandemic, state crimes of “explicit omission” could describe the gross negligence of the Trump Administration that had been informed in advance and chose not to act, failed to adequately inform the US about the situation, and failed to take the necessary steps towards preventing social harm. While other countries also botched their initial response to the COVID-19 pandemic (e.g., Brazil, Mexico, the United Kingdom), the most egregious offender was, until January 2021, President Donald J. Trump, whose refusal to take the virus seriously caused thousands of preventable deaths.

To explicate, months before the outbreak of COVID-19, President Trump had been informed that “mass casualties and economic devastation from the new coronavirus” were on their way (Al Jazeera 2020). The Trump Administration, however, did not heed these official warnings. On February 28, 2020, President Trump accused Democrats of using the coronavirus as a “hoax” to damage him and his administration (Franck 2020). On March 9, 2020, two days after the Governor of the State of New York declared a state of emergency following a severe spike in cases, President Trump continued to claim that COVID-19 was nothing more serious than the common flu (BBC 2020). Moreover, President Trump refused to use the test kits adopted by the World Health Organization (WHO) and preferred that the Center for Disease Control (CDC) develop a new test instead (Dwyer 2020). By the time the CDC had an approved test, the virus had already been in the country for two weeks and had already spread widely (Fink and Baker 2020). In late 2020, the Trump Administration blocked a publication from the CDC which outlined how to reopen public places safely over concerns that the guidance would slow down efforts to reboot the economy quickly (Dearen and Stobbe 2020). Overall, reports from April 2020, in the early days of the pandemic, where taking action was most crucial, reveal that the Trump Administration publicly downplayed the coronavirus at least eleven times (CNN 2020). In fact, in an interview between President Trump and journalist Bob Woodward, the President Trump stated, “I wanted to always play it down. I still like playing it down because I don’t want to create a panic” (NPR 2020).

At this point, it is clear that President Trump’s remarks, including the suggestion that ingesting bleach could be a good remedy for COVID-19 (BBC 2020), contradicted scientific evidence and data arriving from international organizations, had an adverse impact on the public’s understanding of COVID-19 from the very beginning, and failed to protect the population from illness and death. If the Trump Administration had trusted the reports that it had received, followed the guidance of the previous administration, communicated a science-based message to the public and state and local leaders, and adopted the international test kit, containment would have happened much more quickly, and fewer individuals would have fallen ill or lost their lives.

As of February 2021, the death toll in the US has surpassed 500,000 people (five times the number registered in the first draft of the article in May). President Trump’s lack of acknowledgment of the severity and potential danger of the illness can also be deemed responsible for the ongoing national debate and confusion regarding social distancing and face coverings. President Trump spent numerous press briefings mocking reporters for wearing a face mask in public despite advising citizens to adhere to CDC guidelines, which recommend wearing face coverings (Johnson 2020).

A core aspect of “criminal selectivity” is how neither the mainstream media nor the criminal justice system perceived the lack of the Trump Administration’s early intervention, the sharing of misinformation, or the mockery of protective measures in COVID-19 as *crimes* or *harms*, even though people died as a result of these acts and omissions. Just a quick look at the headlines of the US’s most widely read online news sources confirms that public opinion of his actions and inactions has not deemed them to be *criminal* despite the amount of social harm caused. As Koehler (2020) puts it, “It will take a particularly strong case with particularly provable links to Trump’s refusal to dispatch medical equipment or test kits to hot spots in places like New York City or Detroit to bring Trump personally to justice, either criminally or civilly. That will be a tough sell, as they say in litigation circles, even before open-minded judges and juries.”

Koehler’s (2020) statement suggests that Trump will likely benefit from “under-criminalization”—the absence or minimization of the punitive treatment of behaviors committed by those individuals in positions of power. Such harmful inaction has not impacted all people in the same way. Rather, it has resulted in “selective victimization”: Latinos and African-Americans have experienced death tolls that are twice as high as that of the Caucasian population in the US (Mays and Newman 2020). This is likely because those two populations are *over-represented* among the “essential” workers who are on the frontline and exposed to the virus daily. Indeed, as much as 75% of shop employees, drivers, and care workers belong to racial minorities (Mays and Newman 2020). These socioeconomic factors emerge from what might be regarded as “historical state crimes”—crimes that have been at the core of the creation of the state and an intrinsic element of nation-building throughout history. Slavery and segregation represent two examples in the US and have shaped contemporary racism and selective victimization. In the past, the poor and people of color suffered from exploitation, discrimination, and a lack of access healthcare. Today, many minority populations in the US still cannot obtain adequate medical care, increasing their rates of morbidity and mortality, in general, and making them more vulnerable to unattended chronic diseases that aggravate COVID-19 symptoms, in particular (Fouad et al. 2020). In addition, such populations often live in conditions or environments in which maintaining the recommended social distance and washing hands—two “simple” ways of reducing the spread of COVID-19—represent serious challenges.

Sex and race are also important when exploring “selective victimization”: women of color have been disproportionately impacted by the number of job losses since the beginning of the pandemic in the US because they are overrepresented in service sectors, which have been impacted by shutdowns (Micklas 2020). Meanwhile, women, in general, have been overwhelmed with the responsibilities of both work and caregiving at home, and the lack of public policy designed to compensate these disparities could amount to a state crime of omission (Micklas 2020).

COVID-19 has also exposed another state crime of omission resulting in sex-based “selective victimization”—the lack of comprehensive sex-based violence public policies. Home isolation and the disruption of social and community networks have given rise to an alarming increase in sex-based violence during the pandemic. For example, in the first quarter of 2020, the city of Chicago experienced a 12% increase in domestic violence calls to the police (Bosman 2020)—adding further support to the proposition that President Trump’s negligence in preventing the spread of the disease and his failure to implement (or even endorse) policies to protect the poor, racial minorities, and women, who are particularly affected by the pandemic, could be framed as state “crime of omission.”

### Crimes of Globalization

“Crimes of globalization” are “those demonstrably harmful policies and practices of institutions and entities that... by their very nature occur within a global context” (Rothe and Friedrichs 2015: 26). They occur, “in part, by the policies of neoliberalism and the actions of international financial institutions such as the World Bank and the International Monetary Fund and, in part, by the competitive needs of global markets and capital accumulation” (Barak 1991: 8). These crimes deserve particular attention as they involve the most powerful global interests influencing or making decisions that oftentimes result in negative consequences for the masses.

The United Nations (UN) and the WHO have been working to address several types of global crises arising in the face of COVID-19, including a lack of data and information sharing between countries and access to essential medical supplies and personal protective equipment, as well as more general humanitarian aid. Nonetheless, mid-pandemic, President Trump cut funding for the WHO. This may constitute a crime of globalization because Trump’s decision was a state-based decision that resulted in a reduced budget of the main global actor addressing the pandemic on a global scale, and which might have eventually led to countless lives lost worldwide. Richard Horton, editor-in-chief of *The Lancet*, took matters a step further, writing that Trump’s decision regarding the WHO was “a crime against humanity” and that “every scientist, every health worker, every citizen must resist and rebel against this appalling betrayal of global solidarity” (Davidson 2020).

Another decision made by the Trump Administration, which may constitute a crime of globalization in the context of the COVID-19 pandemic, was its refusal to lift sanctions against Iran. The Trump Administration claimed that humanitarian aid was exempt from sanctions and, therefore, any issues with receiving the aid was the fault of Iran’s government. It cannot be denied, however, that US “secondary sanctions on financial institutions and companies that do business with Iran have made it nearly impossible for Iran to buy items like ventilators to treat coronavirus patients” (Fassihi 2020). Months before the pandemic swept the globe, Human Rights Watch (2019) explained that US sanctions “have largely deterred international banks and firms from participating in commercial or financial transactions with Iran, including for exempted humanitarian transactions, due to the fear of triggering US secondary sanctions on themselves.” In short, Iranians were denied access to essential medical care due, at least in part, to these sanctions. It is essential that we consider Trump’s actions and inactions here through the lens offered by the notion of “crimes of globalization.”

Interestingly, the concept of “crimes of globalization” reveals that “selective victimization” can also be analyzed at the state level. In the abovementioned examples, US sanctions and secondary sanctions have threatened the lives of Iranians seeking essential aid and services for survival, while the US’s withdraw of funding to the WHO has had a direct negative impact on the poor nations that are most dependent on the organizations’ interventions. Considering the global power structure, it is unlikely that the US will encounter any repercussions for the adverse effects of its decisions regarding Iran and the WHO.

### State-Corporate Crimes and Harms

“Essential crimes” are perpetrated not only by state and international organizations but also by corporations in collaboration with state agents. These actions have been described as “state-corporate crimes,” which Kramer and Michalowski (1990:3) define as “illegal or socially injurious actions that occur when one or more institutions of political governance pursue a goal in direct cooperation with one or more institutions of economic production and distribution.” Building on this earlier work, Kramer and Michalowski (2006) expose how those in positions of political and economic power perpetrate the most harmful actions and frequently operate in collaboration.

Even though state-corporate crimes are present around the world, the Trump Administration purposely and systematically pursued these offenses as part of an overall program aimed at “rolling back or weakening regulations designed to protect workers, consumers, the environment, and general public health from *known* corporate harms” (Michalowski and Brown 2020: 113 (emphasis in the original)). Moreover, “these rollbacks constitute[d] the most far-reaching effort to free corporate capital from regulatory restraint since the onset of corporate regulation at the dawn of the twentieth century” (Michalowski and Brown 2020: 113).

With COVID-19, state-corporate crime has become widespread (Jones 2020; Okeson 2020) and its harmful effects more dreadful, particularly those perpetrated by the pharmaceutical industry in the US (Griffin and Miller 2011). During the pandemic, the collusion of “Big Pharma” and the Trump Administration could be observed in the rollback of regulations for price-gauging and profiteering coupled with participation in insider trading (Friedrichs and Vegh Weis 2021). Particularly striking is how the Trump Administration managed the information related to potential vaccines. The objective seems to have been to increase profits for corporations and their stakeholders rather than to protect public health: “[i]n late June [2020], a San Francisco company called Vaxart announced that the Trump administration had selected it to develop a coronavirus vaccine. The value of stock options distributed to company insiders just weeks before increased six-fold. Stock options held by Vaxart’s CEO went from $4.3 million to more than $28 million” (Reich 2020). In a similar case, President Trump announced a US$765 million loan to the Eastman Kodak Company, the photography company, to engage in the manufacture or ingredients used in pharmaceuticals, despite the company lacking an arm or subsidiary involved in such activities. Just before this information went public, “Kodak had handed its board of directors 240,000 stock options, and just the day before had given its CEO [Jim Continenza] 1.75 million stock options” (Reich 2020).

Another example of state-corporate crime involves the US Food and Drug Administration (FDA), one of the main regulatory bodies responsible for promoting public health, which made the bold decision to award Gilead Sciences, Inc., the American biopharmaceutical company, full approval for the use of their drug, Remdesivir, to treat COVID-19, despite data from trials completed by the WHO questioning the efficacy of the drug (Cohen and Kupferschmidt 2020). The FDA also awarded Gilead Sciences a priority review voucher, which can be used in the future to speed the review and therefore approval of another drug. In addition, Gilead Sciences has the option to sell this voucher to another company, which could be worth around US$100 million (Brennan 2020). As a further demonstration of this state-corporate crime, Trump’s 2017 financial disclosure forms, which were presented to the United States Office of Government Ethics, revealed that he earned between US$100,001 and US$1 million from the sale of shares in Gilead Sciences, Inc. (Tyko 2020).

It is also necessary to explore how “selective victimization” associated with these state-corporate crimes disproportionally affect disadvantaged people and communities. In the pre-pandemic times of 2019, about 58 million people in the US reported the inability to afford pharmaceutical medicine and 34 million people reported personally knowing someone who had died as a result of lack of access to adequate medical treatment (Lerner 2020). Ironically, it was taxpayers who contributed US$99 million to the development of Remdesivir, and yet the treatment may be unaffordable for many Americans considering that cost estimates for a five-day treatment course for the privately insured will be US$3,120 (Lupkin 2020a, b). Indeed, the treatment cost for the uninsured remains unclear but will probably be higher. This situation is unsettling considering that an estimated 27.5 million people in the US do not have health insurance according to a study of health insurance coverage in the US (Berchick, Barnett and Upton 2019). This population includes informal workers, undocumented immigrants, and other vulnerable populations, such as the homeless or unemployed.

Another example of a state-corporate crime is the previously mentioned state-corporate denial of the scope of the pandemic, despite extensive scientific evidence to the contrary, which placed the lives of millions of people at stake. State-corporate crime can “consist[]... of corporate propaganda built around lies and deceptions masquerading as science, which is then disseminated by ideological and political forces in conservative think tanks, industry trade associations, the corporate media and some government officials” (Kramer 2012: 47). One of Trump’s largest misinformation advocates has been the Fox News Channel, whose audience is at heightened risk from COVID-19 because the average age of viewers is sixty-five. Interviews with experts on the channel have been surrounded by false claims coming from politicians, such as Trump, making it nearly impossible for viewers to understand which of the interviewees can be trusted to provide fact-based and data-backed information. The Fox News Channel allows these false claims to be stated as if they were facts rather than to allow them to be rebutted by experts, which seems intended to “mislead a vulnerable public about risks and harms,” according to an open letter sent to Fox Corporation’s chairman, Rupert Murdoch, by journalists and teachers begging for truth during the pandemic (Gitlin 2020). Misinformation furthers the reach and intensifies the dangers of the pandemic, but Trump is great for Murdoch’s ratings and Murdoch provides a crucial platform for Trump’s dangerously misleading information—the perfect recipe for a state-corporate crime that leaves older adults, the most vulnerable population during this pandemic, selectively victimized.

### Corporate Crimes

Without needing to rely on the state, corporations, by themselves, also participate in activities that they deem to be essential to their success and which we have referred to as “essential crimes.” Within the context of the COVID-19 pandemic, the extent of corporate crime has been particularly striking. Corporations have been increasing their profit throughout the pandemic while exposing their workers to unsafe working conditions. For example, Amazon increased the value of its shares by 40% in April 2020 (Soper 2020) and, at the same time, fired unionized warehouse employees in the US who were fighting for safe working conditions amidst the global pandemic. Two corporate employees were fired just for organizing a virtual event in which warehouse employees would be able to discuss working conditions with tech employees at Amazon. Another employee was suspended and then terminated for providing a coworker with a 24-hour hotline to report concerns about the warehouse shuttle service, for requesting that management implement social distancing measures for employees’ clock-in stations, and for recommending paid leave for part-time workers (Sainato 2020). This type of retaliation against employees that pressured Amazon to protect the health and safety of its workers, who were deemed *essential* during the pandemic, is just one example of a corporate crime going unpunished in the US.

“Selective victimization” also becomes apparent when we look closely at the pay rate offered by Amazon and the demographics of warehouse employees. According to 2017 data collected by the National Employment Law Project, 54% of warehouse employees in California were Latinx, 9.5% were Asian-American, and 9% African American (Tung and Berkowitz 2020). These workers were deemed *essential*, yet were awarded just a US$2.00/hour pay increase during the pandemic in exchange for risking their lives in the potentially unsafe warehouse working conditions, aggravated by the revocation of unlimited paid time off at the end of April 2020 (O’Brien 2020).

Several other large US companies, which were not considered *essential* businesses, forced employees to violate stay-at-home orders and report to work, thus unlawfully exposing them to the threat of contracting the virus with deadly consequences, in what might be regarded as an “essential crime” by corporations. Some of the companies accused of this were the call center that administers surveys called TenPoint Complete, the craft store chain, Michaels, and GameStop, the video game and consumer electronics store (Davenport 2020).

Along the same lines, throughout the pandemic, some private hospitals have been unable to provide adequate personal protective equipment for frontline healthcare workers, have ordered them to not wear their masks while on the job, and have fired them for speaking out, exposing them to the illness (Davenport 2020). This corporate crime not only causes harm to those working in the profession but also to the patients that these potentially infected employees are treating. For example, a nurse at Select Specialty Hospital, owned by Select Medical, was working with patients with acute or chronic respiratory disorders—a population that is at high-risk for serious complications caused by COVID-19 (Davenport 2020). Despite her hospital having medical protective equipment available, healthcare workers were not allowed to wear these masks in the common areas of the hospital to avoid scaring other patients. Laura Moreno, the nurse in this predicament, explained to *The Washington Post*, “I was told if I wanted to wear a mask, I would not be working there. So, I said I’m not willing to put my life at risk, and my contract was terminated” (quoted in Davenport 2020). Lauri Mazurkiewicz, a nurse at Northwestern Memorial Hospital, was prohibited from using an N95 mask that she brought into work herself and was instead instructed to use the less protective surgical masks that were provided by the hospital. She then wrote to some coworkers warning them of the ineffectiveness of surgical masks in protection against COVID-19 and was fired from her position (Davenport 2020).

No less harmful have been the actions of big corporations in increasing the prices of vital drugs and health-related supplies, including masks, gloves, and sanitizers without relation to production or commercialization costs. Massachusetts received reports of pharmacies charging US$30 for eight ounces of hand sanitizer and New York City issued 550 violations for price gouging on items in high demand (Levenson 2020). About forty states have laws against price gouging, yet many of the laws were just recently “activated by emergency declarations issued by their governors in response to the coronavirus pandemic” (Levenson 2020). Some sellers withheld masks to manipulate the market and later charged outrageous prices. As a result, healthcare facilities and citizens were forced to buy these essential products at exorbitant amounts that far exceeded their normal retail price. According to an article in the *Wall Street Journal*, which reviewed federal contracting data, the US government placed more than US$110 million in mask orders at inflated prices with unproven vendors that claimed to have masks and offer quick delivery. The average cost per mask purchased was US$6.00—roughly six times the nonpandemic price (Maremont 2020).

Overall, from mainstream media to the health industry and the pharmaceutical complex, corporations have been profiting from the pandemic at the expense of the health and the lives of employees and the general population. While these entities have abused the notion of “essential,” as this article has suggested, such harmful acts and omissions are often deemed *essential* to the powerful to remain powerful. In other words, these socially injurious acts and omissions—these “essential crimes”—are part and parcel of a global neoliberal capitalist order. The COVID-19 pandemic has helped illuminate just how *essential* these crimes and harms are to the perpetuation of the status quo by the powerful.

## “Essential Punishments” in Times of COVID-19

Modern history has responded to crime with state-based and prison-centered punishment (Vegh Weis 2017), but the COVID-19 pandemic has called into question what types of punishment are “essential” to society. Many states and counties in the US have approved the early release of people behind bars, based on the belief that their imprisonment is not *essential* for public safety. Should this reasoning set the tone for a reconsideration of why those imprisonments had ever been imposed in the first place? Should states have waited until a pandemic to make decisions regarding the lives of people imprisoned in inhumane conditions despite those people not posing a threat to public safety?

In April 2020, the Federal Bureau of Prisons reported that it had released 1,300 people to home confinement in response to COVID-19, although this is a small fraction of the 170,000 individuals in federal custody (Brennan Center for Justice 2020). At the state level, California has granted early release to 3,500 incarcerated individuals and Los Angeles County has released close to 25% of its jail population (Brennan Center for Justice 2020), while Iowa has released 482 incarcerated people (Brennan Center for Justice 2020). Kentucky Governor Andy Beshear (D) signed an executive order releasing nearly 900 people detained in the commonwealth’s prisons (Brennan Center for Justice 2020). Maryland has released nearly 200 people from its juvenile detention centers and New Mexico Governor Michelle Lujan Grisham (D) signed an executive order releasing some of the state’s incarcerated individuals (Brennan Center for Justice 2020). New York State announced the release of 1100 people incarcerated across the state who were imprisoned because of parole violations, while in New York City, fifty-one young prisoners were released from Rikers Island and sixty-five set free from a prison in Westchester County (Brennan Center for Justice 2020). Ohio has recommended the release of 141 incarcerated people in minimum security prisons, and Oklahoma is commuting the prison sentences of more than 450 people behind bars, while Pennsylvania has taken steps that could lead to the release of between 1500 and 1800 “non-violent, at-risk” individuals (Brennan Center for Justice 2020). Washington state will commute the sentences of up to 950 incarcerated people who are considered to be part of a vulnerable population (Brennan Center for Justice 2020). These decisions are consistent with the statement issued by the UN Sub-Committee on Prevention of Torture (2020), which called for governments worldwide to reduce the numbers of incarcerated people.

Early releases due to COVID-19 might seem like a sign of empathy on the part of decision-makers, but they are actually a necessary reaction to the fact that prisons are unsanitary depositories that lack the means to prevent the spread of the virus through social distancing and sanitization. The fact that correctional healthcare systems cannot deal with massive outbreaks of coronavirus in prison has been the rationale for early release, not a sudden bout of clemency or leniency. Despite the figures listed above, 98% of those who have sought early release on compassionate grounds have had their requests rejected and are still suffering in unhealthy prison conditions (Blakinger and Neff 2020). Indeed, as of December 2020, one in every five people imprisoned in the US had COVID-19 (Schwartzapfel et al. 2020). We must also remember that “over-criminalization” has resulted in African-Americans, Latinos, and the poor being disproportionately held in prisons. Latinos and African–Americans are stopped by police, convicted and incarcerated at a much higher rate than Whites (Alexander 2010), meaning that prisoners with COVID-19 are more likely to be members of these minority groups.

The situation in the immigration enforcement system is not much better. An investigation by *The New York Times* and The Marshall Project revealed how Immigration and Customs Enforcement (ICE) became a spreader of COVID-19 (Kassie and Marcoloni 2020). The detention centers for immigrants are unsanitary and cramped, making social distancing nearly impossible; protective gear is rarely available. In addition, the Trump Administration pressured countries to hold deportees who, because they had been infected with the virus, spread it upon arrival at their destinations (Kassie and Marcoloni 2020).

It is worth considering that the COVID-19 pandemic has shown *all of us* how difficult isolation is and the threat it poses to our mental and physical well-being. Indeed, we spent several weeks or months—depending on the region—in confinement, albeit with many advantages: 1) we were often in the comfort of our own homes; 2) we knew that our friends and neighbors were experiencing the same confinement; 3) we believed that the confinement would be short-lived; and 4) we had access to the internet to be able to continue to socialize digitally, exercise, research, stay up-to-date with current events, and entertain ourselves. Despite all these advantages, we were afflicted by feelings of imprisonment and suffocation, the loss of sense of time, a lack of agency, and loneliness. All the while, we held fast to the belief that we needed to maintain balance and not let any previously made life progress slip away. The WHO (2020) reported that the pandemic “has disrupted or halted critical mental health services in 93% of countries worldwide … bereavement, isolation, loss of income and fear are triggering mental health conditions or exacerbating existing ones.” While confinement in one’s home is far different from imprisonment in a correctional facility, the glimpse of isolation that we were provided during COVID and its lasting harmful effects might create an opportunity to discuss jail and prison as punishment for crime.

We must ask ourselves: what would it be like, then, to be in this situation of confinement, but in a drab, hostile, foreign place, without creature comforts, for months or years, at the mercy of a third party (e.g., prosecutors, judges, prison staff, parole boards) on which we have little influence, without access to the internet, almost no contact with our loved ones, and very few daily activities to keep us occupied? Can we imagine that this would improve our lives in any way? Although incarceration and state-imposed isolation as a response to COVID-19 are incommensurable, sheltering-in-place did grant us the opportunity to appreciate the confinement is not a positive experience. Moreover, many of the benefits that we have been able to enjoy during self-isolation, such as cooking, spending time with the family, or learning a new skill, are not available in prison. Shall our personal experiences during the pandemic set the basis for a discussion about confinement as a suitable response to crime and an eventual debate on prison reform or abolition?

Overall, the fact that public safety was not threatened by early releases, that states themselves recognized the deplorable conditions of prisons that made them dreadful places in the context of a pandemic, and the worldwide experience of the iatrogenic effects of isolation should call into question whether imprisonment should still exist. It should force us to consider if imprisonment, itself, regardless of the pandemic, is a form state harm. It should compel us to pay renewed attention to abolitionist literature, which has, for decades, been pointing to the limitations of criminal law as a means to solve problems (see, e.g., Coyle and Schept 2018). Isolation due to COVID-19 should provide us with a chance to rethink prison as an unsuitable solution to crime. And it should force us to examine whether prison is *essential* to protect us from those who do harm.

## Conclusion

This article has argued that the COVID-19 pandemic has afforded us the opportunity to consider what is genuinely *essential* in our lives. In so doing, it has allowed us to understand how certain harmful acts and omissions are essential to the perpetuation of power by the powerful—what we have called “essential crimes.” In addition, the COVID-19 pandemic has offered a unique occasion to rethink what is *essential* in terms of punishment. Focusing on the pandemic in the US as a case study, we have suggested that crimes of the powerful and reduction (and, perhaps, abolition) of prisons are the *essential* challenges of the present.

Ten years ago, Friedrichs (2011) put forth the notion of a “prospective criminology”—one that could try, to the extent possible, to prevent harm from happening in the first place. Academic work generally engages with past facts because it relies on data that need to be processed, posing an obstacle for the study of prospective crimes. Given our experience with COVID-19, we might reconsider how to embrace Friedrichs’ challenge. As Friedrichs states, the idea is to draw upon criminological knowledge to contribute uniquely and significantly to the understanding of crimes of the powerful as a way to develop prevention methods or effective responses. The world has become increasingly global and, while the COVID-19 virus, itself, has not been a crime of the powerful, the pandemic has created opportunities for crimes of the powerful and has exposed how certain “essential crimes” perpetuate imbalances and cause harm. Hopefully, we can use this experience to put our research to the service of building awareness of some of the prospective crimes that might take place so that we can protect that which is really *essential*.
